# How do economic crises affect migrants’ risk of infectious disease? A systematic-narrative review

**DOI:** 10.1093/eurpub/ckv151

**Published:** 2015-08-28

**Authors:** Alexander Kentikelenis, Marina Karanikolos, Gemma Williams, Philipa Mladovsky, Lawrence King, Anastasia Pharris, Jonathan E. Suk, Angelos Hatzakis, Martin McKee, Teymur Noori, David Stuckler

**Affiliations:** 1 Department of Sociology, University of Cambridge, Cambridge, UK; 2 European Observatory on Health Systems and Policies, London School of Hygiene and Tropical Medicine, London, UK; 3 LSE Health, London School of Economics and Political Science, London, UK; 4 Department of International Development, London School of Economics and Political Science, London, UK; 5 European Centre for Disease Prevention and Control (ECDC), Stockholm, Sweden; 6 Medical School, University of Athens, Athens, Greece; 7 Department of Sociology, University of Oxford, Oxford, UK

## Abstract

**Background:** It is not well understood how economic crises affect infectious disease incidence and prevalence, particularly among vulnerable groups. Using a susceptible-infected-recovered framework, we systematically reviewed literature on the impact of the economic crises on infectious disease risks in migrants in Europe, focusing principally on HIV, TB, hepatitis and other STIs. **Methods:** We conducted two searches in PubMed/Medline, Web of Science, Cochrane Library, Google Scholar, websites of key organizations and grey literature to identify how economic changes affect migrant populations and infectious disease. We perform a narrative synthesis in order to map critical pathways and identify hypotheses for subsequent research. **Results:** The systematic review on links between economic crises and migrant health identified 653 studies through database searching; only seven met the inclusion criteria. Fourteen items were identified through further searches. The systematic review on links between economic crises and infectious disease identified 480 studies through database searching; 19 met the inclusion criteria. Eight items were identified through further searches. The reviews show that migrant populations in Europe appear disproportionately at risk of specific infectious diseases, and that economic crises and subsequent responses have tended to exacerbate such risks. Recessions lead to unemployment, impoverishment and other risk factors that can be linked to the transmissibility of disease among migrants. Austerity measures that lead to cuts in prevention and treatment programmes further exacerbate infectious disease risks among migrants. Non-governmental health service providers occasionally stepped in to cater to specific populations that include migrants. **Conclusions:** There is evidence that migrants are especially vulnerable to infectious disease during economic crises. Ring-fenced funding of prevention programs, including screening and treatment, is important for addressing this vulnerability.

## Introduction

The economic crisis that has afflicted Europe since 2008 has been linked to several infectious disease outbreaks, especially among vulnerable populations. These include localized epidemics of human immunodeficiency virus (HIV) among injecting drug users in Greece and Romania,^1–4^ and, in Greece, the re-emergence of locally acquired malaria between 2009 and 2012,^5^^,^^6^ and, more recently, an increase in tuberculosis (TB) notifications.^7^^,^^8^ At the same time, some policy makers^9^^,^^10^ and news outlets^11^^,^^12^ have attributed the incidence of infectious disease to increased migration, noting that some migrant populations have higher rates of TB, HIV and other infectious diseases.

In reality, migrants are often initially healthier overall than the host country population,^13^ although they are at higher risk of carrying latent forms of some infectious diseases. Some groups may also be disproportionately at risk of specific infectious diseases due to increased exposure to risk in their country of origin, during the migration journey and as a consequence of adverse socioeconomic conditions in the destination country.^14–16^ Yet, the links between migration, the economic crisis and recent outbreaks remain unclear, and are further complicated by the fact that increases in HIV and TB have been concentrated in non-migrants.^17–20^

Although there is now extensive literature documenting the association between economic turmoil in Europe and population health,^18–27^ so far the inter-relationship between migration, economic crisis and communicable disease incidence has received less attention. The pathways involved are complex, nonlinear and characterized by variable lag periods.^28^^,^^29^ One way to conceptualize them, using the example of TB, is the susceptible-infected-recovered (SIR) model, which considers the magnitude of susceptible populations, transmissibility of disease and the availability and effectiveness of treatment.^28^ Here, we build on this model to present an iterated SIR framework that captures the dynamic nature of the migration process (Box 1). If, for instance, migrant workers are made redundant, they might become homeless, and opt to return to their home countries, thereby reducing the size of the susceptible population and thus TB incidence in the host country in the short term. Similarly, a budget cut to TB treatment programmes for migrants could increase death rates in the short term, so lowering disease prevalence and associated spread, while potentially exacerbating longer-term epidemic trajectories for both local and immigrant populations.^30^

Box 1 Economic crises and the susceptible-infected-recovered conceptual framework

Conceptualizing how crises can affect infectious disease among migrants presents important analytic challenges. First, economic crises lead to changes in policy – e.g. in prevention programmes – that can affect the transmission of infectious disease.^18^^,^^20^^,^^21^^,^^28^^,^^58,61,91^ Second, migration itself leads to changes in the composition of populations: as people move between countries, so can infectious disease profiles change. Third, many infectious diseases manifest as long-term latent infections before becoming clinical cases, therefore complicating analyses of the effects of economic crises. Finally, evidence on the interactions between economic problems and migratory flows is mixed:^92,93^ crises can lead to declines in migration by those seeking employment, but leave other types of migration unaffected (e.g. for family reasons, refugees or environmental migrants).^94^ At the same time, crises can intensify population movements within the host country or free-movement zones (such as the EU/EEA), as people migrate in search of employment or other sources of support.^94,95^

To model such diverse dynamics, we build on the SIR framework, that traces infectious disease risks from the population susceptible to the disease, to those infected, and finally to those who recover or die.^28^^,96^ The figure below presents an iterated SIR framework that is compatible with the nature of the migratory process. For parsimoniousness, we assume that migration ends as a migrant reaches the host country, but that need not be the case: the model can be extended to capture further population movements to other host countries or a return to the country of origin.

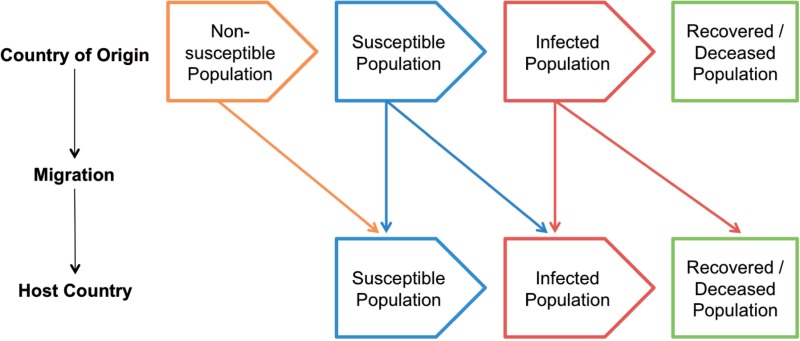

The iterated SIR framework enables a number of distinct possibilities to be examined. A migrant may not have been susceptible to infectious disease, but could become susceptible and infected in the host country. Or a migrant may have already been susceptible in the country of origin, but could become infected in the host country or during the migration process. Similarly, a migrant may have been infected in the country of origin and remain infected in the host country. Extending the model enables further scenarios; for example, a migrant may have become susceptible in the country of origin, then became infected in the host country, and subsequently returned to the country of origin to gain access to treatment.

This article reviews the available literature on the impact of the economic crisis on infectious disease among migrant populations in Europe. While we searched for literature without restrictions on a range of infectious diseases, we only found relevant evidence for TB, hepatitis, HIV and other sexually transmitted infections (STIs), and our analysis is focused on these. First, we present background information on the geography of infectious disease risks among migrants in Europe, as well as the main transmission routes. Second, we outline the methods employed in the study. Third, through the lens of the SIR framework, we review the literature to determine how crisis-related pathways affect infectious disease incidence, screening and treatment for migrants in Europe. We conclude by discussing the policy implications of these findings, and emerging directions for future research.

### Background: burden of infectious diseases in migrant populations in Europe

Population movements have transformed EU member-states over the past two decades, encompassing both migration from outside the EU and within it.^31^ The early years of the crisis (2007–2010) saw a decline in migratory flows from outside the EU, which reversed in 2011.^32^ At the same time, high unemployment rates in the EU’s periphery and the removal of mobility restrictions for citizens of new member-states accelerated intra-EU migration.^32^^,^^33^ As of January 2013, 33.5 million residents of EU-27 countries were born outside the EU-27 (6.9% of the population), and 17.3 million persons resided in a different EU-27 country than the one they were born in.^34^

Detailed information on migration trends in Europe is not matched by systematic data on the health of these migrants,^35^ and their characteristics: they comprise a broad set of sub-groups with heterogeneous backgrounds (e.g. asylum seekers from war-torn areas, students moving within the EU and economic migrants) who have varying risk factors and health profiles. The main sources of data on infectious disease among migrant populations are the European Centre for Disease Prevention and Control’s (ECDC) European Surveillance System (TESSy) and population surveys from individual countries. Table 1 summarizes available surveillance data on several infectious diseases in migrant populations.

**Table 1:** Infectious disease in migrant populations in Europe

Disease	Data from	Reporting countries	Migrant status variable used	Reported cases with data on migrant or import status		Most cases^a^ originating abroad in …	Least cases^a^ originating abroad in …
				Total	Of which reported cases among foreign-born population (%)		
HIV	2012	30	Country of birth; country of nationality or region of origin	25 297	35.9% (45% of which originating from Sub-Saharan Africa; 17% from Latin America and the Caribbean; 15% from Central and Eastern Europe)	Luxembourg (77%)	Bulgaria, Croatia, Hungary, and Romania (all <10%)
						Sweden (67%),	
						Norway (61%) UK (57%)	
TB	2011	29	Country of birth^b^	72 334	25.8%	Sweden (89.4%), Norway (87.8%)	Romania (<1%), Poland (<1%)
Hepatitis B	2011	18	Imported	6662	52.6%	Sweden (96.1%)	Estonia (0%)
Hepatitis C	2011	17	Imported	12 111	8.3%	Ireland (55.1%)	Czech Republic (0%)
Syphilis	2010	12	Country of birth	9991	7.3% (13% of which originating from Asia, and 11% from Africa)	Finland (46.5%)	Romania (0%)
Gonorrhoea	2010	11	Country of birth	8992	11.1% (46% of which originating from another-EU/EEA European country, and 18% from South America)	France (26%)	Romania (0%)
						Cyprus (17%)	
						Netherlands (15%)	

a:Data provided here are for illustration purposes only; cross-country comparisons are challenging due to different surveillance systems used by member states. There is wide variation in case definitions used for some diseases (hepatitis B and C), data completeness and definition of migrant relevant variables.

b:Except Austria, Belgium, Hungary and Poland who used ‘citizenship’ for classification.

*Sources* HIV: ECDC, WHO Regional Office for Europe. 2013. *HIV/AIDS Surveillance in Europe 2012*. Stockholm: European Centre for Disease Prevention and Control.

TB: ECDC. 2013. *Tuberculosis Surveillance and Monitoring in Europe 2013*. Stockholm: European Centre for Disease Prevention and Control.

Hepatitis B and C, syphilis, gonorrhoea, measles and rubella: ECDC. 2014. *Assessing the burden of key infectious diseases affecting migrant populations in the EU/EEA*. Technical Report. ECDC: Stockholm.

Epidemiological reports published since the onset of the crisis shed light on the varying patterns of infectious disease in European populations, including migrants. Between 2007 and 2012, 39.9% of HIV cases were in migrants.^1^ The majority of migrant cases were from sub-Saharan Africa (54.3%), with high proportions from Latin America (12.2%), Western Europe (9.5%) and central Europe (6%). The number of new migrant HIV cases diagnosed during the period rose slightly, with increases among migrants from Latin America, Central and Eastern Europe but decreases among migrants from sub-Saharan Africa.^1^

For TB, approximately one quarter of notified cases in 2012 were among people of non-EU/EEA origin,^36^ of which two-thirds were in individuals from Asia or Africa and 6% from the former Soviet Union.^37^ Although the total TB notification rates in the EU/EEA have declined over recent decades, the contribution of foreign-born subjects to the total pool of TB has been increasing each year in many member-states, representing a challenge for TB control programs and a public health concern.^37^^,^^38^

Surveillance data compiled by the ECDC also report a higher burden of chronic hepatitis B infections in migrants than in the native population.^39^ In a meta-analysis of data on chronic infection in migrants to Europe, the highest prevalence of hepatitis B virus (HBV) among migrant and refugee populations was in migrants from high HBV-endemicity regions in East Asia and the Pacific (11.3%) and sub-Saharan Africa (10.3%).^40^

While the surveillance data provide a general picture of the overall trends, there are important limitations. Despite efforts to harmonize data collected by national surveillance systems on migrant-specific variables such as ‘country of birth’ in recent years, the type and quality of data collected still varies between EU/EEA countries and reporting on migrant-specific variables is poor for the majority of diseases, with the exceptions of HIV and TB.

Finally, a rise in the incidence of Chagas disease in Europe in the last decade, primarily in Belgium, France, Italy, Spain, Switzerland and the United Kingdom, has been attributed to increased migration from endemic areas in Latin America.^41^ However, it is difficult to obtain an accurate picture of prevalence and incidence, as Chagas disease is not systematically monitored or reported and most population or hospital-based studies were undertaken only in Spain.^42^

In line with the varying patterns of infectious disease incidence, the modes of transmission for individual diseases can differ in migrants and natives, and between sub-groups of migrants, depending on region of origin and other factors.^17^ Box 2 summarizes the latest available information on modes of transmission and the limitations of the data. The impact of the economic crisis on these patterns is, however, poorly understood. This study seeks to address this gap in knowledge.

Box 2 Changing modes of transmission in recent years

In 2012, sub-Saharan Africa was identified as the origin of 13.8% of all HIV diagnoses in the EU/EEA, 35.0% of heterosexually acquired infections and 38.3% of mother-to-child transmissions,^1^ consistent with other studies in Spain^97^ and the UK.^98^ Data from 2012 show that the majority of new cases of HIV in sub-Saharan African migrants were attributed to heterosexual transmission, while the majority of new cases (whether among the native-born population or migrants from Latin America, Eastern Europe and East Asia) were in men who have sex with men (MSM).^17^ In addition, recent evidence from the United Kingdom suggests that a growing number of migrants are being infected after arrival to the country: 2011 data reveal that 48% of heterosexuals born abroad with HIV contracted the virus after arrival to the UK.^99,100^ Further evidence suggests an increased rate of post-migration HIV transmission among some immigrant groups, as reported in Norway and the UK.^101^

Surveillance data on STIs also suggest differences in the mode of transmission between migrants and non-migrants.^17^ Between 2000 and 2010, cases of syphilis in migrants were most likely to be acquired through heterosexual transmission (57%), unlike in non-migrant cases, most of which were transmitted through MSM contact (65%).^17^ Between 2004 and 2010, cases of gonorrhoea among migrants were mostly acquired through heterosexual transmission, whereas heterosexual and MSM transmission accounted for broadly similar shares of cases in non-migrants.^17^ In 2011, the majority of cases of hepatitis B in migrants were classified as mother-to-child-transmission (72.7%). Hepatitis B in non-migrants was more likely to be acquired through heterosexual transmission or injecting drug use.^17^

There is mixed evidence on the transmission of TB cases in migrant groups and how it compares with natives in the EU/EEA. In Germany, a low probability of TB transmission between migrants and natives has been reported, with authors noting how fear of migrants increasing the risk of TB was unjustified.^102,103^ In Denmark, TB transmission was 2.5 times more likely to occur from non-migrants to migrants than *vice versa*.^102^ Data from 2002 to 2003 from Barcelona suggest that transmission among Spanish-born and migrant populations, and bidirectional transmission between Spanish-born and foreign-born populations were issues of concern.^104^

## Methods

Given the paucity of studies examining the impact of economic crises on infectious diseases among migrants, we selected search criteria to maximize the number of studies included. Migrants were defined as the foreign-born population regardless of their country of origin; this definition excludes short-term movements of individuals (e.g. tourists or business travellers). We adopted a broad view of ‘economic crisis,’ including economic downturns, associated changes in unemployment rates, and policy responses (austerity measures or structural reforms). All infectious diseases were included in the review. The search focused on European countries, defined as those in the European Union and European Economic Area (EU/EEA; see Box 3).

Box 3 Search terms

For the PubMed search, we used the following list of key words:

1. Infectious Disease Terms
  – Infectious disease*, infection, communicable disease*, vaccine preventable diseases, zoonotic infection*, imported tropical disease*
  – Chagas, American trypanosomiasis, enteric fevers: enteric fever*, salmonella typhi, salmonella, paratyphi, typhoid fever, paratyphoid fever, Hepatitis B, HBV, viral hepatitis, Hepatitis C, HCV, HIV/AIDS, measles, rubella, malaria, plasmodium, STI* (congenital syphilis and gonorrhea), Tuberculosis, TB, West Nile virus
2. Economic crisis terms
  – Cris*, economic cris*, financial cris*, austerity, adjustment, bailout, downturn, recession, employment, unemployment
3. Study population: migrants
  – Migration, immigration, emigration, migrant*, immigrant*, emigrant*, foreign*, asylum seeker*, refugee*, irregular, citizen*, citizenship, nationalit*
4. Study setting: EU/EEA
  – Austria, Belgium, Bulgaria, Croatia, Cyprus, Czech Republic, Denmark, Estonia, Finland, France, Germany, Greece, Hungary, Iceland, Ireland, Italy, Latvia, Liechtenstein, Lithuania, Luxembourg, Malta, Netherlands, Norway, Poland, Portugal, Romania, Slovakia, Slovenia, Spain, Sweden, United Kingdom, Great Britain, England, Wales, Scotland, EU, EU/EEA, Europe*, European Union, European Economic Area

### Search strategy

We searched four electronic databases (PubMed/Medline, Web of Science, Cochrane Library and Google Scholar) and 18 websites of key organizations, as well as scanning reference lists of papers. The database search strategy was conducted using various combinations of key words for the four main axes of interest: infectious diseases, study population (migrants), the economic crisis and its implications (austerity measures, rising unemployment), and the study setting (EU/EEA). In addition, using web searches, we also investigated grey literature, including reports by specialized agencies, non-governmental organizations (NGOs) and news items.

The search terms included ‘infectious’, ‘infection’, ‘communicable’, ‘vaccine preventable’, as well as a wide range of specific diseases. The crisis-related terms included ‘cris*’, ‘austerity’, ‘downturn’, ‘recession’, ‘employment’, ‘unemployment’ and ‘adjustment.’ A number of search terms were included for migration: ‘migration’, ‘immigration’, ‘emigration’, ‘migrant*’, ‘immigrant*’, ‘emigrant*’, ‘foreign*’, ‘asylum seeker*’, ‘refugee*’, ‘irregular’, ‘citizen*’, ‘nationalit*.’ The names of all 31 EU/EEA countries as well as ‘Europe*’ were included as the study setting terms.

To maximize the number of included studies, we conducted the literature searches in two parts, focusing on: the economic crisis and migrant health; and the economic crisis and infectious disease, respectively. The method used was a narrative synthesis, in order to map proximal and intermediate pathways and identify hypotheses for subsequent research.^43^ This approach was appropriate given that data are limited and often haphazardly situated in a large corpus of literature. For instance, some studies link the incidence of infectious disease to economic hardship, whereas other studies document that the latter disproportionately affects migrants.^14^^,^^44^^–^^46^ We synthesize such findings in order to yield nuanced understandings of the relationships of interest.

### Selection criteria

Articles were considered for inclusion if they: (i) were descriptive and analytic observational studies, experimental studies, reviews, systematic reviews and meta-analyses; (ii) were published between January 2007 and February 2014; (iii) were published in English; and (iv) included data from the EU/EEA countries.

Eligibility was initially assessed by screening all identified papers and reports based on title and abstract. The full text was then obtained for all selected articles and a second screening performed to determine final eligibility. Data relevant to the study objectives were retrieved.

### Results of the literature search

First, the database search on the association between economic crises and migrant health initially identified 653 studies, with 68 studies selected for full-text review and seven meeting the final inclusion criteria. In addition, 14 items of grey literature were identified. Second, the database search on the association between economic crises and infectious disease initially identified 480 studies, with 33 studies selected for full-text review and 19 meeting the final inclusion criteria. In addition, eight items of grey literature were identified. Appendices 1 and 2 present PRISMA flowcharts for these literature searches. Only the most relevant studies are cited in this article.

## Economic crisis and infectious disease among migrants: pathways

In this section, building on the SIR framework presented in Box 1, we review literature on how the economic crisis and its consequences have affected infectious disease transmissibility among migrants in Europe by risk factor (S to I), as well as the implications of changes in availability of screening and treatment (I to R).

### Transmissibility of disease

#### Unemployment

The changing socioeconomic environment has potentially important consequences for infectious disease among migrants. Since the onset of the crisis, unemployment rates in many European countries have soared: the Euro-area unemployment rate rose from 7.5% in 2007 to 12% in 2013, with some countries – notably, Greece and Spain – well above that rate.^47^ Migrants from outside the EU have been disproportionately affected by job loss.^44^ Unemployment and the consequent sharp declines in income can lead to destitution, malnutrition and deterioration in living conditions (e.g. overcrowded homes or homelessness). In turn, these have been associated with increased risk of infection or exacerbation of existing infections, as they are linked to increased exposure to other risk factors.^14^^,^^45,46,48,49^ For example, several studies have shown how increased unemployment, impoverishment and associated rises in homelessness in Athens have increased the size and interconnectivity of networks of persons who inject drugs.^26^^,^^46,50^ This could mean that migrants are also at increased risk of infection,^14^ although no studies were identified to confirm this. Further information on this topic is provided in the following section.

#### Drug use

The changed socioeconomic environment can affect risk factors for drug use. The economic crisis may encourage a shift to cheaper drugs and, if these are administered intravenously and/or induce risky behaviour, they may affect the incidence of infectious disease.^51^^–^^53^ For example, the European Monitoring Centre for Drugs and Drug Addiction (EMCDDA) notes a ‘fledgling trend’ in crystal methamphetamine smoking in Southern Europe,^54^ with reports of its use among migrants in Greece. Media stories have linked the introduction of the drug to the crisis, as it is easily produced and cheaper than alternatives such as heroin.^55^ Derivative harms include earlier initiation to injecting and greater injection risk behaviours, which are linked to blood-borne virus transmission.^56,57^

Austerity measures have also affected programmes designed to prevent infections resulting from drug use. In Greece, public health prevention programs suffered large cuts during 2008–2011, during a time of increased need during the 2011 HIV outbreak among injecting drug users in Athens.^20^^,^^21^ During the outbreak, needle exchange programmes, condom distribution and opioid substitution treatment were improved, although remained at sub-optimal levels.^50^ These trends are likely to affect all drug users adversely, but some evidence suggests that austerity measures were also specifically linked to increased infectious disease among migrants. While the majority of new HIV infections were among Greek citizens, the newly diagnosed cases of HIV in Greece included some migrants and it was found that new HIV-1 strains circulating in the major heroin-producing area of Afghanistan had been introduced into needle-sharing networks of susceptible drug injectors in Athens.^26^ Beyond Greece, EMCDDA has raised broader concerns about the impact of austerity measures on drug policy, with eight countries reducing drug-related public spending since the onset of the crisis.^51,52^ As noted above, such cuts can affect infectious disease incidence among migrants.

### Prevention and treatment

#### Health service delivery

Wide-ranging austerity measures in a number of European countries have often resulted in cuts to public healthcare, health workers and/or treatment interventions,^28^^,^^45,48,58,59^ with migrants at particular risk of not receiving adequate healthcare.^60^ In a scoping study of infectious disease experts early in the crisis, over two-thirds of respondents speculated that health services for vulnerable groups – especially migrants – would deteriorate as a consequence of the crisis, while 85% of respondents reported that no policies or programmes were being implemented to mitigate negative effects of the recession.^61^ One study attributed Greece’s weakened ability to deal with the HIV outbreak, mentioned above, to austerity measures that restricted public facilities hiring new medical staff.^3^ Spain has also reportedly cut government funding for public health policies in relation to HIV/AIDS, a move anticipated to exacerbate risks to undocumented migrants.^62,63^ In addition, cuts in Italy have reduced central transfers to regions and local government for services for migrants and other welfare measures, potentially reducing access to infectious disease-related services.^64^

Infectious disease control may be further impaired if budget cuts reduce the ability of health sectors to scale-up prevention and control activities quickly in the event of disease outbreaks.^26^^,^^58,59^ Further to the evidence presented above, a survey of health policy responses to the financial crises in Europe found that other countries (e.g. Latvia and Estonia) reported cuts to public health budgets, although it was not always clear whether communicable disease prevention and control programmes were directly affected.^58^

At the same time, the economic crises in Europe and their fallout have also affected health service provision by the non-governmental sector. Data on NGO-financed health expenditure provided by the OECD^65^ are haphazard and marked by changes in methodology and breaks in the data, and – therefore – is not used here. However, NGOs in Southern Europe report initiating interventions to respond to unmet need for health care. In Greece, global NGOs like Médecins Sans Frontières^66^^–^^68^ and Médecins du Monde (MDM)^69,70^ have scaled up their operations, with some of their interventions targeted especially at migrant groups. Other NGOs gained access to *ad hoc* funds for specific interventions (e.g. for combating the spread of HIV or newly arriving migrants and asylum seekers), which in turn allowed them to deploy more services.^71^ In Spain, NGOs have also been becoming more prominent in providing healthcare to those migrant groups excluded from the health system. For example, even though the Spanish chapter of MDM experienced falls in revenue and a decline in overall funding for operations, its spend on programs targeting migrants increased by 45% between 2010 and 2011.^72^ In addition, in some instances, Spanish local authorities collaborate and/or subcontract services with NGOs to provide some services to undocumented migrants.^73^ Such service provision may strengthen infectious disease prevention, yet we could not identify studies to corroborate this.

#### Entitlement

Structural reforms to health systems can also affect infectious disease prevention and treatment, insofar as they reduce coverage in terms of migrants’ entitlements to services. A recent review of European experiences highlights how migrants face challenges in obtaining sexual and reproductive health services, often as a result of unclear legal provisions.^74^

Two studies from Spain conducted during the crisis period compared access with services or treatment of migrants with that of the non-migrant population. A comparison of the uptake of HIV testing among Spaniards and Latin-American migrants showed that the latter were more likely to get tested in 2008–2011 than non-migrants.^75^ A survey of migrants and the native population in Catalonia examined perceptions of continuity of treatment in the Catalan public healthcare system. The survey found that, in 2010, migrants and native-born population experienced similar levels of managerial (care coherence) and informational (patient information transfer) continuity of treatment.^76^ While this evidence from Spain appears encouraging, it refers to the period prior to the introduction of a Royal Decree in 2012 changing the basis of entitlement for access to public healthcare services. The new system reportedly excludes around 500 000 undocumented migrants from accessing comprehensive healthcare service,^75,77^^–^^79^ but given that the introduction of this decree is very recent and was again modified subsequent to the period covered by this review, we could not identify studies documenting its effects on the incidence of infectious disease among migrants.

#### User charges

Cost-shifting by increasing user charges can also increase barriers to receiving healthcare for vulnerable groups, including migrants.^58^ Increased user charges and/or co-payments have been noted in at least 13 member states,^18^^,^^59^ although it is not clear how this may have affected access to infectious disease prevention and treatment services specifically. A descriptive study of access to health services for migrants in Greece in 2013 found that 62% (144/231) of immigrants in Greece who responded reported unmet health need, while 53% (122/231) of those surveyed reported they had major difficulties accessing health services. The main reason for not being able to do so was financial cost and long waiting lists.^80^ Such barriers to accessing services can have implications for the treatment of infectious disease, although these are not identified in that study.

## Conclusion

Our study demonstrates that the economic crises in Europe and their consequences for health systems and society have potentially or actually increased the risks associated with infectious disease hazards. Our knowledge is fragmented and inconclusive, but available information suggests that some groups of migrants have worsening health risks, and that budget cuts clearly constrained public health responses. During an economic crisis some types of migrants are likely to experience deteriorating socioeconomic situation and living conditions, while risk factors and disease susceptibility are likely to increase. However, the extent to which these changes will translate into actual infections depends on the adoption and implementation of public health responses, by governments and civil society, as well as other factors.

We note that our study has important limitations, some of which have already been mentioned. First, there were few studies explicitly investigating the link between economic crises and migrant health. This reflects several methodological challenges. One is the lack of a universal definition of migrants across Europe. EU member-states variously may classify migrants according to nationality, country of birth, residency or duration of stay,^44^ confounding cross-country analyses. In addition, many European countries do not routinely collect or disaggregate infectious disease data according to migrant status.^17^^,^^81^ Further, migrant status is only one way by which foreign-born populations may be at increased risk of infectious disease. Other mobile groups – e.g. tourists, business people or short-term contract workers – may also be at risk of infectious disease but data limitations for these populations are even more severe.

Second, data are often delayed. While economic data are made available rapidly, usually with a delay of a few months at most, morbidity and mortality data often become available only with a 2–5-year delay.^18^^,^^64^ Although there may be information on specific outbreaks that have attracted attention (e.g. the HIV outbreak in Greece), the broader effects on a European level may take longer to be fully revealed. Moreover, the effects of crises are also lagged and may take several years or decades to become apparent in morbidity and mortality measures.^82^

Third, this study has elaborated several pathways linking economic crises in Europe to infectious disease risks among migrants. However, infectious disease incidence and prevalence is also affected by less proximal, ‘upstream’ crisis-related factors, such as politics, legal environments or structural inequalities.^83,84^ This set of factors involves long causal chains that are often hard to study, but can eventually translate into micro-level determinants. For instance, governments’ political orientations may result in immigration policy reforms affecting the conditions in detention and initial reception centres across Europe or health-service entitlements of asylum applicants.

These limitations point to a clear need for more research on the effects of economic crises on migrant health, including infectious disease among these vulnerable populations. In addition, future research can adopt a wider perspective and examine the relationship between migration and asylum policies of European countries and infectious disease screening, prevention and treatment programs for migrants.

These findings have important implications for infectious disease control programmes in Europe. First, policies that prevent and control infectious disease need to be maintained, and – if appropriate – strengthened. Combating outbreaks increases the costs falling on countries already under fiscal stress, and is likely to cost more than scaling up prevention measures. To this end, ensuring adequate and timely access to health services for migrant populations is of central importance.^85,86^ For example, undocumented migrants comprise a key vulnerable population as they often have very limited access to prevention and treatment services and, in some countries, policies are reducing this further.^87^^–^^89^ Second, the various organizations involved in the migration and health policy areas would do well to enhance collaboration and information exchange in this area. Finally, screening practices for infectious disease among migrants remain inconsistent across Europe, highlighting the importance of the ongoing work by ECDC to develop evidence-based guidance and foster coordination.^90^

As the evidence of the broader health impact of the crisis and the associated policy response is amassing, countries undergoing economic change should take account of what evidence does exist and invest in infectious disease prevention and control. The imperative for further work on these issues is urgent, as it can enable policy makers to devise appropriate and timely evidence-based responses.

## Supplementary data

Supplementary data are available at *EURPUB* online.

## Funding

The study was funded by the European Centre for Disease Prevention and Control (ECDC). D.S. is funded by a Wellcome Trust Investigator Award.

*Conflicts of interest*: None declared.

## References

[1] ECDC, WHO Regional Office for Europe. HIV/AIDS surveillance in Europe 2012. Stockholm, European Centre for Disease Prevention and Control, 2013.

[2] Paraskevis D Nikolopoulos G Tsiara C HIV-1 outbreak among injecting drug users in Greece, 2011: a preliminary report. Euro. Surveill. 2011;16:1–4. 10.2807/ese.16.36.19962-en 21924120

[3] Pharris A Wiessing L Sfetcu O Human immunodeficiency virus in injecting drug users in Europe following a reported increase of cases in Greece and Romania, 2011. Euro. Surveill. 2011;16:1–5. 22172301

[4] Nikolopoulos GK Fotiou A Kanavou E National income inequality and declining GDP growth rates are associated with increases in HIV diagnoses among people who inject drugs in Europe: a panel data analysis. PLoS One 2015;10:e0122367. 25875598 10.1371/journal.pone.0122367 PMC4398461

[5] Danis K Baka A Lenglet A Autochthonous plasmodium vivax malaria in Greece, 2011. Euro. Surveill. 2011;16:pii=19993. 22027375

[6] Danis K Lenglet A Tseroni M Malaria in Greece: historical and current reflections on a re-emerging vector borne disease. Travel Med. Infect. Dis. 2013;11: 8–14. 23434287 10.1016/j.tmaid.2013.01.001

[7] Spala G Epidemiological data for tuberculosis in Greece. Athens: Hellenic Centre for Disease Control and Prevention (KEELPNO), 2013.

[8] Odone A Tillmann T Sandgren A Tuberculosis among migrant populations in the European Union and the European economic area. Eur. J. Public Health 2015;25:506–12. 25500265 10.1093/eurpub/cku208 PMC4440450

[9] Loverdos A Statement at the high level meeting on AIDS. United Nations. 2011 Available at: http://www.unmultimedia.org/tv/webcast/2011/06/greece-h-e-andreas-loverdos-2011-high-level-meeting-on-aids-plenary-meeting.html (28 March 2014, date last accessed).

[10] Davies L Beppe Grillo sparks controversy with image of blacked-up politician. The Guardian. 2014 Available at: http://www.theguardian.com/world/2014/sep/03/italian-comedian-activist-beppe-grillo-controversy-blacked-up-image-politician (7 December 2014, date last accessed).

[11] Thomas JM Sorry but migrants will STILL abuse the NHS. Daily Mail. 2013 Available at: http://www.dailymail.co.uk/debate/article-2531479/Sorry-migrants-STILL-abuse-NHS-Department-Health-research-shows-16-charges-recovered.html (28 March 2014, date last accessed).

[12] Slack J Toll of mass migration on UK life. Daily Mail. 2013 Available at: http://www.dailymail.co.uk/news/article-2355208/Toll-mass-migration-UK-life-Half-Britons-suffer-strain-places-schools-police-NHS-housing.html (28 March 2014, date last accessed).

[13] Mladovsky P A framework for analysing migrant health policies in Europe. Health Policy 2009;93:55–63. 19586680 10.1016/j.healthpol.2009.05.015

[14] Gushulak B Pace P Weekers J Migration and health of migrants. In: Koller T, editor. Poverty and Social Exclusion in the WHO European Region: Health Systems Respond. Copenhagen: WHO Regional Office for Europe, 2010: 257–81.

[15] Semenza JC Giesecke J Intervening to reduce inequalities in infections in Europe. Am. J. Public Health 2008;98:787–92. 18381991 10.2105/AJPH.2007.120329 PMC2374832

[16] Rechel B Mladovsky P Devillé W Migration and health in the European Union: an introduction. In: Rechel B Mladovsky P Devillé W Rijks B Petrova-Benedict R McKee M, editors. Migration and Health in the European Union. Maidenhead: Open University Press, 2011: 3–13.

[17] ECDC. Assessing the burden of key infectious diseases affecting migrant populations in the EU/EEA. Stockholm, European Centre for Disease Prevention and Control, 2014.

[18] Karanikolos M Mladovsky P Cylus J Financial crisis, austerity, and health in Europe. Lancet 2013;381:1323–31. 23541059 10.1016/S0140-6736(13)60102-6

[19] Stuckler D Basu S The Body Economic: Why Austerity Kills. New York: Basic Books, 2013.

[20] Kentikelenis A Karanikolos M Reeves A Greece's health crisis: from austerity to denialism. Lancet 2014;383:748–53. 24560058 10.1016/S0140-6736(13)62291-6

[21] Kentikelenis A Karanikolos M Papanicolas I Health effects of financial crisis: omens of a Greek tragedy. Lancet 2011;378:1457–8. 21988763 10.1016/S0140-6736(11)61556-0

[22] De Vogli R Marmot M Stuckler D Excess suicides and attempted suicides in Italy attributable to the great recession. J. Epidemiol. Commun. Health 2013;67:378–9. 10.1136/jech-2012-201607 22859517

[23] Navarro V The social crisis of the Eurozone: the case of Spain. Int. J. Health Serv. 2013;43:189–92. 23821901 10.2190/HS.43.2.a

[24] Gili M Roca M Basu S The mental health risks of economic crisis in Spain: evidence from primary care centres, 2006 and 2010. Eur. J. Public Health 2013;23:103–8. 23132877 10.1093/eurpub/cks035

[25] Modrek S Stuckler D McKee M A review of health consequences of recessions internationally and a synthesis of the US response during the great recession. Public Health Rev. 2013;35:1–33.

[26] Paraskevis D Nikolopoulos G Fotiou A Economic recession and emergence of an HIV-1 outbreak among drug injectors in Athens metropolitan area: a longitudinal study. PLoS One 2013;8:e78941. 24265730 10.1371/journal.pone.0078941 PMC3827120

[27] Economou C Kaitelidou D Kentikelenis A The impact of the financial crisis on health and the health system in Greece. In: Maresso A Mladovsky P Thomson S, editors. Economic Crisis, Health Systems and Health In Europe: Country Experiences. Copenhagen: WHO Regional Office for Europe/European Observatory on Health Systems and Policies, 2015:103–42. 28837306

[28] Suhrcke M Stuckler D Suk JE The impact of economic crises on communicable disease transmission and control: a systematic review of the evidence. PLoS One 2011;6:e20724. 21695209 10.1371/journal.pone.0020724 PMC3112201

[29] Peckham R Contagion: epidemiological models and financial crises. J. Public Health 2014;36:13–7. 10.1093/pubmed/fdt083 23965642

[30] Reeves A Basu S McKee M Social protection and tuberculosis control in 21 European countries, 1995–2012: a cross-national statistical modelling analysis. Lancet Infect. Dis. 2014;14:1105–12. 25303845 10.1016/S1473-3099(14)70927-2

[31] United Nations. International migration and development (database reference: POP/DB/MIG/Stock/Rev.2013). United Nations Department of Economic and Social Affairs (Population Division). 2013. Available at: http://esa.un.org/unmigration/TIMSA2013/migrantstocks2013.htm?mtotals (30 March 2014, date last accessed).

[32] OECD. International Migration Outlook 2013. Paris: OECD Publishing, 2013.

[33] Holland D Paluchowski P Geographical labour mobility in the context of the crisis. Eur. Employ. Observ. 2013 Available at: http://www.niesr.ac.uk/sites/default/files/publications/ESDE-SynthesisPaper-June2013-Final.pdf. (17 August 2015, date last accessed).

[34] Eurostat. Migration and migrant population statistics. 2015. Available at: http://ec.europa.eu/eurostat/statistics-explained/index.php/Migration_and_migrant_population_statistics#Foreign_and_foreign-born_population (8 June 2015, date last accessed).

[35] Rechel B Mladovsky P Ingleby D Migration and health in an increasingly diverse Europe. Lancet 2013;381:1235–45. 23541058 10.1016/S0140-6736(12)62086-8

[36] ECDC. Tuberculosis Surveillance and Monitoring in Europe 2013. Stockholm: European Centre for Disease Prevention and Control, 2013.

[37] Hollo V Amato-Gauci A Kodmon C Tuberculosis in the EU and EEA/EFTA countries: what is the latest data telling us? Euro. Surveill. 2009;14:1–4. 19317981

[38] Abubakar I Stagg HR Cohen T Controversies and unresolved issues in tuberculosis prevention and control: a low-burden-country perspective. J. infect. Dis. 2012;205(Suppl 2):S293–300. 22448025 10.1093/infdis/jir886

[39] ECDC. Hepatitis B and C Surveillance in Europe, 2006–2011. Stockholm, 2013.

[40] Rossi C Shrier I Marshall L Seroprevalence of chronic hepatitis B virus infection and prior immunity in immigrants and refugees: a systematic review and meta-analysis. PLoS One 2012;7:e44611. 22957088 10.1371/journal.pone.0044611 PMC3434171

